# Accuracy Versus Predominance: Reassessing the Validity of the Quasi-Steady-State Approximation

**DOI:** 10.1007/s11538-025-01451-z

**Published:** 2025-05-16

**Authors:** Kashvi Srivastava, Justin Eilertsen, Victoria Booth, Santiago Schnell

**Affiliations:** 1 Department of Mathematics, University of Michigan, Ann Arbor, MI 48109 USA; 2 Mathematical Reviews, American Mathematical Society, 416 4th Street, Ann Arbor, MI 48103 USA; 3 Department of Biological Sciences and Department of Applied and Computational Mathematics and Statistics, University of Notre Dame, Notre Dame, IN 46556 USA

**Keywords:** Singular perturbation, Quasi-steady-state approximation, Michaelis–Menten reaction mechanism, Timescale separation

## Abstract

The application of the standard quasi-steady-state approximation to the Michaelis-Menten reaction mechanism is a textbook example of biochemical model reduction, derived using singular perturbation theory. However, determining the specific biochemical conditions that dictate the validity of the standard quasi-steady-state approximation remains a challenging endeavor. Emerging research suggests that the accuracy of the standard quasi-steady-state approximation improves as the ratio of the initial enzyme concentration, $e_0$, to the Michaelis constant, $K_M$, decreases. In this work, we examine this ratio and its implications for the accuracy and validity of the standard quasi-steady-state approximation as compared to other quasi-steady-state reductions in its proximity. Using standard tools from the analysis of ordinary differential equations, we show that while $e_0/K_M$ provides an indication of the standard quasi-steady-state approximation’s asymptotic accuracy, the standard quasi-steady-state approximation’s predominance relies on a small ratio of $e_0$ to the Van Slyke-Cullen constant, *K*. Here, we define the predominance of a quasi-steady-state reduction when it offers the highest approximation accuracy among other well-known reductions with overlapping validity conditions. We conclude that the magnitude of $e_0/K$ offers the most accurate measure of the validity of the standard quasi-steady-state approximation.

## Introduction

The Michaelis–Menten (MM) reaction mechanism, a cornerstone of enzyme kinetics, describes the irreversible catalysis of a substrate, *S*, into product, *P*, by an enzyme, *E*:

1 $$\begin{aligned} S + E \mathop {\rightleftharpoons }\limits ^{k_1}_{k_{-1}} C \xrightarrow {k_2} E + P \end{aligned}$$

where *C* represents an enzyme-substrate intermediate complex (Michaelis and Menten 1913). The simplest form of the mass action equations modeling the dynamics of the substrate, complex, enzyme, and product concentrations (*s*, *c*, *e*, and *p*, respectively) is a two-dimensional system of ordinary differential equations (ODEs):

2a $$\begin{aligned} {\dot{s}}&= -k_1(e_0-c)s+k_{-1}c, \end{aligned}$$

2b $$\begin{aligned} {\dot{c}}&= \;\;\; k_1(e_0-c)s-(k_{-1}+k_2)c, \end{aligned}$$

with ${\dot{e}}=-{\dot{c}}$, ${\dot{p}}=k_2c$, where “$\dot{\phantom {x}}$” denotes differentiation with respect to time, *t*. The constant, $e_0$, is the total enzyme concentration, a conserved quantity.

Closed-form solutions to (2) are unattainable due to quadratic nonlinearities. Consequently, reduced equations approximating the long-time dynamics of (2) are often employed to both elucidate the kinetics of (1) and serve as a forward model for parameter estimation from in vitro progress curve experiments via nonlinear regression (Schnell and Maini 2003; Stroberg and Schnell 2016; Choi et al. 2017). The standard quasi-steady-state approximation (sQSSA) to the mass action equations associated with the MM reaction mechanism is a well-known and extensively studied reduced model in biochemical kinetics (Schnell and Maini 2003; Schnell 2014). Simply put, the sQSSA is a reduced system that approximates the depletion of substrate:

3 $$\begin{aligned} {\dot{s}} = -\frac{k_2e_0s}{K_M+s}, \quad s(0)=s_0, \end{aligned}$$

where $K_M = (k_{-1}+k_2)/k_1$ is the Michaelis constant and $k_2\,e_0$ is the limiting rate. The Michaelis constant is composite of the equilibrium constant $K_S=k_{-1}/k_1$ and the Van Slyke-Cullen constant $K=k_2/k_1$, and can be expressed as $K_M = K_S + K$. We assume initial conditions for (2) lie on the s-axis, i.e., $(s,c)(0)=(s_0,0)$. Under this assumption, we can equip (3) with the initial condition $s(0)=s_0$.^1^

Geometric singular perturbation theory provides mathematical justification for (3): with $(s,c)(0)=(s_0,0)$, the solution to the first component of the mass action system (2a) converges to the approximation (3) in the limit as $e_0 \rightarrow 0$, provided all other parameters, $k_{-1}, k_2$ and $k_1$, are bounded above and below by positive constants (see, e.g. Goeke et al. 2015; Eilertsen et al. 2024b). For further information on geometric singular perturbation theory, see Kaper and Kaper (2002), Hek (2010), Jones (1995) and Wechselberger (2020).

While the mathematical rationale behind (3) is well-established, applying (3) presents a challenge: determining how small $e_0$ must be to ensure (3) is sufficiently accurate. This assessment is complicated by the relative nature of “small.” $e_0$ is only small compared to another quantity, but what is that quantity? Emerging research (see, e.g. Eilertsen et al. 2023, 2024a, b) suggests that the sQSSA is accurate whenever $e_0 \ll K_M$, or $\varepsilon _{RS} = e_0/K_M \ll 1$, which we refer to as the Reich–Sel’kov condition (Reich and Sel’kov 1974). Thus, the “other” quantity is the Michaelis constant, $K_M$, and the sQSSA is considered *valid*—in the sense of accuracy—when the Reich–Sel’kov condition holds.

Beyond accuracy, it is crucial to determine conditions that ensure the *predominance* of the sQSSA (3). When the reaction unfolds away from the singular limit (i.e., with $0<e_0$), multiple reduced models may be accurate. However, it is not obvious that the Reich–Sel’kov condition guarantees that the sQSSA is the predominant reduction among the nearby Fenichel reductions. Other reduced models may be more accurate^2^ than the sQSSA even when $e_0\ll K_M$. Therefore, identifying the most accurate approximation among the available reduced models with overlapping validity conditions is essential, and this most accurate model should be labeled as the predominant reduction (see Sect. 2 for a better understanding of the class of reduced models considered).

This paper aims to deepen our understanding of the Reich–Sel’kov condition and distinguish between accuracy and predominance concerning the sQSSA (3). To our knowledge, this distinction has not been previously addressed in the literature. Using rigorous phase-plane analysis and methods from ODE analysis, we demonstrate that while the Reich–Sel’kov condition provides a general indication of the sQSSA’s accuracy, it does not ensure its predominance, which requires a more restrictive criterion. We derive this qualifier and challenge the notion that the validity of the sQSSA solely pertains to its accuracy. We argue that validity should encompass both the accuracy and predominance of the sQSSA. In essence, the qualifier establishing the sQSSA’s validity is more restrictive than any previously reported condition.

## Fenichel Theory, TFPV Theory, and Literature Review

This section reviews Fenichel theory and Tikhonov–Fenichel Parameter Value (TFPV) theory, placing the problem within a precise mathematical context. We also examine recent literature on the validity of the sQSSA (3).

### A Brief Introduction of Fenichel Theory

Geometric singular perturbation theory, also known as Fenichel theory (Fenichel 1971/72, 1979), provides the rigorous foundation for deriving the sQSSA. Consider a perturbed dynamical system:

4 $$\begin{aligned} {\dot{x}} = f(x,\varepsilon ),\quad f:\mathbb {R}^n\times \mathbb {R} \rightarrow \mathbb {R}^n \end{aligned}$$

where $\varepsilon \in \mathbb {R}$ is a small parameter close to zero.^3^ If the set of singularities:

5 $$\begin{aligned} S_0:= \{x\in \mathbb {R}^n: f(x,0)=0\} \end{aligned}$$

is a non-empty *k*-dimensional submanifold of $\mathbb {R}^n$ where $0< k < n$, then (4) is *singularly perturbed* (Wechselberger 2020). The rank of the Jacobian with respect to *x*, $D_1f(x,0)$, is constant and equal to $n-k$ along $S_0$.

#### Persistence and Flow

A key result from Fenichel theory states that if $S_0$ is compact and normally hyperbolic—meaning the $n-k$ non-zero eigenvalues of $D_1f(x,0)$ are strictly bounded away from the imaginary axis along $S_0$—then $S_0$ persists. This means an invariant manifold, $S_0^{\varepsilon }$, exists for $\varepsilon \in [0,\varepsilon _0)$. Expressing $S_0^{\varepsilon }$ as a graph over the first *k* coordinates of *x*

6 $$\begin{aligned} S_0^{\varepsilon }:=\{(z,y,\varepsilon )\in \mathbb {R}^k \times \mathbb {R}^{n-k}\times \mathbb {R}: y = h(z,\varepsilon )\}, \end{aligned}$$

where $x:=(z,y)$ and $z\in \mathbb {R}^k,\, y\in \mathbb {R}^{n-k}$, the flow on $S_0^{\varepsilon }$ is satisfies:

7 $$\begin{aligned} {\dot{z}}_i = f_i(z,h(z,\varepsilon ),\varepsilon ),\quad 1\le i \le k \end{aligned}$$

where the $f_i$’s are the component functions of *f*. Equation (7) describes the evolution of *k* independent variables. The evolution of the remaining $n-k$ variables depends on the evolution of *z*:

8 $$\begin{aligned} {\dot{y}} = D_1h(z,\varepsilon ){\dot{z}},\quad D_1h(z,\varepsilon ) \in \mathbb {R}^{(n-k)\times k}. \end{aligned}$$

#### Critical Manifolds and Stability

In singular perturbation theory, the submanifold $S_0$ of stationary points is the *critical manifold*. The persistence of a normally hyperbolic $S_0$ asserts the existence of a normally hyperbolic invariant manifold, $S_0^{\varepsilon }$, and also the persistence of its stable and unstable manifolds, $W^s(S_0)$ and $W^u(S_0)$. Thus, $S_0^{\varepsilon }$ inherits the stability properties as $S_0$.^4^ If $S_0$ is normally hyperbolic and attracting, (7) describes the long-time evolution of (4) because trajectories near $S_0^{\varepsilon }$ contract towards it exponentially.

#### Approximating the Slow Flow

Fenichel’s initial results require explicit knowledge of $S_0^{\varepsilon }$, which is often unavailable in practice. To address this, Fenichel (1979) proposed a method to approximate the flow on $S_0^{\varepsilon }$ without explicitly knowing it. Expanding $f(x,\varepsilon )$ in a Taylor series near $\varepsilon =0$ gives:

9 $$\begin{aligned} {\dot{x}} = f(x,0) + \frac{\partial f(x,\varepsilon )}{\partial \varepsilon }\Bigg |_{\varepsilon =0}\cdot \varepsilon + {\mathcal {O}}(\varepsilon ^2) =: f_0(x) + \varepsilon f_1(x) + {\mathcal {O}}(\varepsilon ^2). \end{aligned}$$

If $S_0$ is normally hyperbolic^5^ and attracting, a splitting exists:

10 $$\begin{aligned} T_x\mathbb {R}^n \cong \mathbb {R}^n = \ker D_1f(x,0) \oplus \;\text {image} \;D_1f(x,0) \end{aligned}$$

for all $x\in S_0$, where $T_xS_0 \cong \ker D_1f(x,0)$ for all $x\in S_0$. Projecting the right-hand side of (9) onto $T_xS_0$ approximates the dynamics on $S_0^{\varepsilon }$. This projection is achieved using the operator $\pi ^s:\mathbb {R}^n \rightarrow \ker D_1f(x,0)$; see Goeke and Walcher (2014) and Wechselberger (2020) for details on the construction of $\pi ^s$. The resulting *n*-dimensional dynamical system (with *k* independent variables) is:

11 $$\begin{aligned} x' = \pi ^s f_1(x)|_{x\in S_0} \end{aligned}$$

where “$\phantom {x}'$” denotes differentiation with respect to the slow timescale, $\tau = \varepsilon t$. The dynamics on $S_0^{\varepsilon }$ determined by (7) converges to (11) as $\varepsilon \rightarrow 0$. Equation (11) is referred to as quasi-steady-state approximation (QSSA) in chemical kinetics.

#### Convergence and Initial Conditions

Solutions to (4) with initial condition $x(0)=(z,y)(0)=(z_0,y_0)$ generally do not converge to the solution of (11) with initial condition *x*(0) unless *x*(0) is sufficiently close to $S_0$. To ensure convergence, both (2) and (11) generally require a modified initial “$\tilde{x}_0$” (see, “Appendix F” for details).

### From Theory to Practice: Tikhonov–Fenichel Parameter Values

Traditional methods for putting mass action equations into perturbation form involve scaling and dimensional analysis, which can be unreliable. A more robust approach considers the mass action equations for a general reaction mechanism:

12 $$\begin{aligned} {\dot{x}} = f(x,p), \quad f:\mathbb {R}^n \times \mathbb {R}^m \rightarrow \mathbb {R}^n \end{aligned}$$

where *p* represents an *m*-tuple of parameters. A point $p^*$ in parameter space is a Tikhonov–Fenichel Parameter Value (TFPV) (Goeke et al. 2015, 2017) if:

1. The set $S_0=\{x\in \mathbb {R}^n: f(x,p^*)=0\}$ is a *k*-dimensional submanifold of $\mathbb {R}^n$ with $0<k<n$, and
2. $S_0$ is normally hyperbolic.

Expanding *f*(*x*, *p*) near $p^*$ in a direction $\rho$, $p=p^* +\varepsilon \rho$, gives^6^

13 $$\begin{aligned} {\dot{x}} = f(x,p^*) + \varepsilon D_2f(x,p^*)\rho , \end{aligned}$$

which matches the form (11). This highlights the need to specify a path in parameter space for taking the limit as $\varepsilon \rightarrow 0$ and $p\rightarrow p^*$.

For the MM system (2), the parameter space is a subset of $\mathbb {R}^4_{\ge 0}$ with $p=(e_0,k_1,k_{-1},k_2)^T$. Here, $p^*:=(0,k_1,k_{-1},k_2)^T$ is a TFPV if $k_1$, $k_{-1}$, $k_2$ are bounded by positive constants. When $p=p^*$, the critical manifold, $S_0$, is the *s*-axis, which is normally hyperbolic and attractive:

14 $$\begin{aligned} S_0:= \{(s,c)\in \mathbb {R}^2_{\ge 0}: c=0\}. \end{aligned}$$

To put (2) into perturbation form, we define a curve $\ell (\cdot )$ that passes through $p^*$

15 $$\begin{aligned} \ell (\varepsilon ) = \begin{pmatrix}0\\ k_1\\ k_{-1}\\ k_2\end{pmatrix} + \varepsilon \begin{pmatrix}\widehat{e}_0\\ 0\\ 0\\ 0\end{pmatrix} = p^* + \varepsilon \rho \end{aligned}$$

where $e_0\mapsto \varepsilon \widehat{e}_0$. This effectively equates “$\varepsilon$” with the magnitude of $e_0$.^7^ The perturbation form of (2) for small $e_0$ is

16 $$\begin{aligned} \begin{pmatrix}{\dot{s}}\\ {\dot{c}}\end{pmatrix} = \begin{pmatrix}(k_1s+k_{-1})c\\ -(k_1s+k_{-1}+k_2)c\end{pmatrix} + \varepsilon \widehat{e}_0k_1 s\begin{pmatrix}-1\\ \;\;\;1\end{pmatrix} \end{aligned}$$

Projecting the right hand side of (16) onto $TS_0$ yields the sQSSA (3):

17 $$\begin{aligned} \begin{pmatrix}{\dot{s}} \\ {\dot{c}}\end{pmatrix} = -\frac{\varepsilon \widehat{e}_0 k_2 s}{K_M+s}\begin{pmatrix}1\\ 0\end{pmatrix} \equiv -\frac{k_2e_0 s}{K_M+s}\begin{pmatrix}1\\ 0\end{pmatrix}. \end{aligned}$$

This derivation implies that the flow on $S_0^{\varepsilon }$ converges to (17) as $e_0 \rightarrow 0$, with other parameters fixed and positive. Furthermore, the solution to the first component of (2) also converges to the first component of (17) since the initial condition $(s_0,0)\in S_0$.

Other TFPV values exist for (2). For instance, with $p^* = (e_0,k_1,k_{-1},0)^T$, the critical manifold becomes

18 $$\begin{aligned} S_0:= \left\{ (s,c)\in \mathbb {R}^2_{\ge 0}: c = \frac{e_0s}{K_S + s}\right\} \end{aligned}$$

and the corresponding Fenichel reduction is the slow product QSS reduction:

19 $$\begin{aligned} \begin{pmatrix} {\dot{s}}\\ {\dot{c}} \end{pmatrix} = -\frac{k_2e_0 s(K_S+s)}{K_Se_0 + (K_S+s)^2}\begin{pmatrix} 1\\ \frac{K_Se_0 - K(K_S+s)}{(K_S+s)^2}\end{pmatrix}. \end{aligned}$$

This reduction differs from (17) and will be analyzed in Sect. 4.

### The Open Problem: Identifying the Predominant Reduction

Just as important as what Fenichel and TFPV theory tell us is what they do *not*. While the theory indicates that (17) approximates (2) as $e_0\rightarrow 0$, it does not specify how small $e_0$ must be for (17) to be valid in practice, where the singular limit is never truly reached. In other words, how small is small enough?

The size of $e_0$ must be considered relative to other parameters. Fenichel theory requires eigenvalue disparity near the critical manifold. For small $e_0$, the ratio of slow and fast eigenvalues near $(s,c)=(0,0)$ is:

20 $$\begin{aligned} \delta :=\frac{e_0}{K_M}\cdot \frac{k_2}{k_{-1}+k_2} + {\mathcal {O}}(e_0^2). \end{aligned}$$

While $\delta \ll 1$ is necessary, it is not sufficient for the accuracy of (17) (Eilertsen et al. 2023). For instance, consider the case where $e_0=K_M$ but $k_2\ll k_{-1}$. This scenario renders small $\delta$, but the tangent vector to the graph of the *c*-nullcline at the origin will not align with the slow eigenvector, which is crucial for long-time accuracy. This misalignment can lead to significant errors in the sQSSA approximation, even when $\delta$ appears to indicate otherwise.

The more restrictive condition $e_0\ll K_M$, introduced in Reich and Sel’kov (1974), has been rigorously proven sufficient to guarantee the accuracy of the sQSSA (Eilertsen and Schnell 2020; Eilertsen et al. 2024a; Kang et al. 2019; Mastny et al. 2007; Eilertsen et al. 2022a). However, this condition does not fully address the issue of predominance. For instance, if $k_{-1}$ is large and $e_0$ is reduced such that $k_1e_0 \ll k_{-1}$ (implying $e_0\ll K_M$), convergence to (17) is not implied. In fact, as $k_{-1} \rightarrow \infty$, the resulting singular perturbation problem yields a trivial QSSA: ${\dot{s}}={\dot{c}}=0$ (see  “Appendix E” for the formal calculation).

**Fig. 1:**
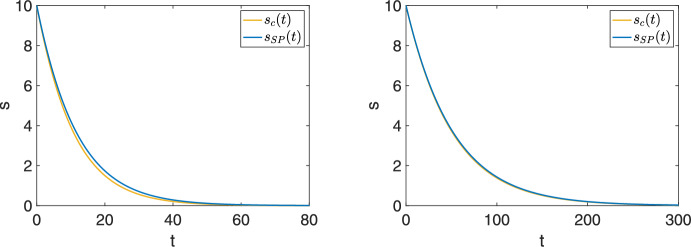
The sQSSA (17) and the slow product QSSA (19) are indistinguishable for large $k_{-1}$. Left: $s_0 = 10$, $c_0 = 0$, $e_0 = 10$, $k_1 = 1$, $k_{-1} = 100$, $k_2 = 1.$ Right: $s_0 = 10$, $c_0 = 0$, $e_0 = 10$, $k_1 = 1$, $k_{-1} = 500$, $k_2 = 1.$ Substrate depletion over time is shown in both panels. $s_c(t)$ is the numerical solution to (17) and $s_{SP}(t)$ is the numerical solution to (19)

This example demonstrates that different TFPVs can share a critical manifold and be close in parameter space. For instance, consider a scenario where $k_1e_0$ and $k_2$ are relatively small compared to $k_{-1}$. In this case, the TFPVs $(0,k_1,k_{-1},k_2)^T$ or $(e_0,k_1,k_{-1},0)^T$ could be quite close to each other. Terminating the approach to one TFPV might inadvertently lead to a point in parameter space that is actually closer to another TFPV.

Numerical simulations (Fig. 1) illustrate this phenomenon. When $k_{-1}$ is significantly large, the trajectories of the system under the sQSSA (17) and the slow product QSSA (19) become virtually indistinguishable. This makes it challenging to definitively determine which reduction is more accurate or appropriate for the given parameter values.

Moreover, the presence of multiple TFPVs introduces ambiguity in assessing the validity of the sQSSA. For example, if $e_0=1$, $k_1=0.1$, $k_{-1}=10$, and $k_2=0.1$, it is unclear whether the system’s behavior is better approximated by the sQSSA associated with the TFPV $(0,k_1,k_{-1},k_2)^T$ or the slow product QSSA associated with $(e_0,k_1,k_{-1},0)^T$. This ambiguity arises because the parameter values do not fall neatly within the domain of a single TFPV, making it difficult to determine which reduction provides the most accurate representation of the system’s dynamics. Thus, we define the *predominance* of a given QSS reduction within the class of QSS approximations derived from Fenichel theory for nearby TFPVs. Established validity conditions, such as the Reich–Sel’kov condition, can often guide us toward identifying TFPVs with overlapping validity conditions, facilitating a robust analysis of predominance. However, in general, determining proximity to different TFPVs remains challenging due to the varying units of the parameters, necessitating a dimensionless indicator.

**Remark**: The approximation of the slow manifold is a well-studied area, with methods extending beyond Fenichel’s reduction theory. For instance, the Fraser iterative method provides approximations for slow manifolds with small curvatures (Fraser 1988; Nguyen and Fraser 1989; Kaper and Kaper 2002). In such cases, incorporating additional iterative terms can enhance the accuracy of the approximations. Furthermore, several studies employ integration-based methods to generate numerical approximations of the slow flow within a specified error tolerance (Davis and Skodje 1999). In our work, we focus on other Fenichel reductions to examine the predominance of the standard QSSA, given that their convergence to the slow flow has been extensively established for the Michaelis-Menten mechanism.

The fact that the magnitude of $e_0/K_M$ alone does not ensure the sQSSA (17) is the *predominant* reduction is a consequence of having to always operate away from the singular limit in a large parameter space. This naturally leads to the question: does there exist a dimensionless parameter whose magnitude not only ensures the accuracy of the sQSSA (3) but also its predominance among other known QSSAs? In other words, can we define a more comprehensive and robust notion of “validity" that encompasses both accuracy and predominance?

## Anti-funnels and the Slow Invariant Manifold

As discussed in Sect. 2, a central challenge in applying Fenichel theory to the MM system is the potential ambiguity arising from different TFPVs sharing the same critical manifold. This can lead to uncertainty about the validity of a specific reduction away from the singular limit. To address this, we turn to qualitative methods that allow us to determine the location of the slow manifold, $S_0^{\varepsilon }$, relative to known curves in the phase-plane. This section introduces the anti-funnel theorem, adapted from Hubbard and West (1995) and following the approach of Calder and Siegel (2008). We outline a strategy to determine when the sQSSA (3) is not only accurate but also predominant.

### [Style2 Style1 Style3 Style3]Definition 1

(*Fences and anti-funnels.*) Consider the first-order differential equation $y' = f(x,y)$ over the interval $x \in I = [a,b)$ where $a<b \le \infty$ and “$\phantom {x}'$" denotes differentiation with respect to *x*. Let $\alpha$ and $\beta$ be continuously-differentiable functions that satisfy

21 $$\begin{aligned} \alpha '(x) \le f(x,\alpha (x)) \; \text {and} \; f(x,\beta (x)) \le \beta '(x) \end{aligned}$$

for all $$x \in I$$.

- The curve $\alpha$ is a *lower fence* and the curve $\beta$ is an *upper fence*.
- The lower and upper fences are *strong fences* if the respective inequalities are always strict.
- The set
  22 $$\begin{aligned} \Gamma := \{(x,y) \;: \; x \in I, \; \beta (x) \le y \le \alpha (x)\} \end{aligned}$$
  is called an *anti-funnel*.^8^ if
  $$\begin{aligned} \beta (x) < \alpha (x) \; \; \text {for all} \; \; x \in I. \end{aligned}$$
- The anti-funnel is *narrowing* if
  23 $$\begin{aligned} \lim \limits _{x\rightarrow b^-} |\alpha (x) - \beta (x)| = 0. \end{aligned}$$

### Theorem 1

Anti-funnel Theorem (Hubbard and West 1995). Consider the first-order differential equation $$y' = f(x,y)$$ over the interval $$I = [a,b)$$ where $$a<b \le \infty $$. If $$\Gamma $$ is an *anti-funnel* with a strong lower fence, $$\alpha $$, and a strong upper fence, $$\beta $$, then there exists a solution *y*(*x*) to the differential equation such that

24 $$\begin{aligned} \beta (x)< y(x) < \alpha (x) \; \; \text {for all} \; \; x \in I. \end{aligned}$$

Furthermore, if $\Gamma$ is *narrowing* and $\frac{\partial f}{\partial y} (x,y) \ge 0$ in $\Gamma$, then the solution *y*(*x*) is unique.

**Fig. 2:**
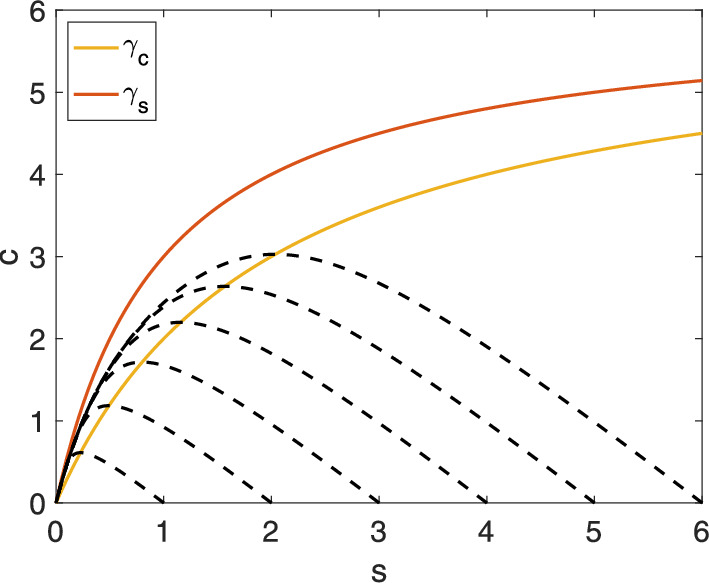
The region, $\Gamma$, between the horizontal nullcline and the vertical nullcline curves is a trapping region on the phase plane. The numerical solutions to the mass action equations (1) for several initial conditions are denoted by black dashed lines. $s_0 \in \{1,2,3,4,5,6\}$, $c_0 = 0$, $e_0 = 6$, $k_1 = 1$, $k_{-1} = 1$, $k_2 = 1.$ The numerical solution of trajectories start on the *s*-axis and eventually enter $\Gamma$

For the MM equations (2), we have:

25 $$\begin{aligned} \frac{\textrm{d}c}{\textrm{d}s} = -\frac{e_0s -(K_M+s)c}{e_0s-(K_S+s)c} =: f(c,s). \end{aligned}$$

The distinguished solution to (25), “$c=y(s)$”, satisfying (24) represents the slow manifold, $S_0^{\varepsilon }$. The challenge lies in finding suitable lower and upper fences, $\alpha (s)$ and $\beta (s)$.

A natural choice for the upper fence is the *c*-nullcline, $\gamma _c$, given by:

26 $$\begin{aligned} \text {Graph}(\gamma _c)=\{(s,c) \in \mathbb {R}^2: c = e_0s/(K_M+s)\} \end{aligned}$$

Considering the biochemically relevant portion of $\mathbb {R}^2$, we focus on $\text {Graph}(\gamma _c) \cap \mathbb {R}^2_{\ge 0}$. This choice is advantageous because it represents the quasi-steady-state variety corresponding to the standard reduction (3), and all phase-plane trajectories starting on the s-axis eventually cross it (see Calder and Siegel 2008; Noethen and Walcher 2007 for a proof of this statement).

The set $\Gamma$ enclosed between the *c*-nullcline and the *s*-nullcline, $\gamma _s$,^9^ is positively invariant:

27 $$\begin{aligned} \Gamma :=\{(s,c)\in \mathbb {R}^2_{\ge 0} \;: \; \gamma _c(s) \le c \le \gamma _s(s)\} \end{aligned}$$

Once a trajectory enters $\Gamma$, it remains within $\Gamma$ as $t\rightarrow \infty$ (see Calder and Siegel 2008; Noethen and Walcher 2007 for a detailed proof). Figure 2 illustrates this behavior.

Constructing a suitable lower fence requires more analysis. The stationary point $(s,c)=(0,0)$ is a node with eigenvalues $\lambda _-<\lambda _+<0$:

28 $$\begin{aligned} \lambda _{\pm } = \frac{k_1}{2}(K_M+e_0)\left( -1\pm \sqrt{1-\frac{4Ke_0}{(K_M+e_0)^2}}\right) . \end{aligned}$$

Under timescale separation in which

29 $$\begin{aligned} \frac{Ke_0}{(K_M+e_0)^2} \ll 1/4, \end{aligned}$$

trajectories eventually approach the stationary point along the slow eigenvector, $v^+$, spanning the one-dimensional subspace:

30 $$\begin{aligned} T_0S_0^{\varepsilon } = \text {span}(v^+) = \{(s,c)\in \mathbb {R}^2: c= ms\}, \end{aligned}$$

with slope *m*:

31 $$\begin{aligned} m= \frac{1}{2k_{-1}}\left( -k_{-1} - k_2 + k_1e_0 + \sqrt{(k_{-1} + k_2 + k_1e_0)^2 - 4k_1k_2e_0}\right) . \end{aligned}$$

Calder and Siegel (2008) leverage this property and define the lower fence:

32 $$\begin{aligned} \alpha _{cs}(s):= \frac{me_0s}{e_0+ms}, \end{aligned}$$

and prove that $S_0^{\varepsilon }$ lies between $\gamma _c(s)$ and $\alpha _{cs}(s)$. Notably, the graph of $\alpha _{cs}$ lies below that of $\gamma _s$. By proving that

33 $$\begin{aligned} \Gamma _{cs}:= \{(s, c) \;: \; s\in I, \gamma _c(s) \le c \le \alpha _{cs}(s)\} \end{aligned}$$

is a narrowing anti-funnel,^10^ Calder and Siegel (2008) prove that the distinguished slow manifold, $S_0^{\varepsilon }$, also lies in $\Gamma$, the region contained between the *c*- and *s*-nullcline.

A key feature of (32) is $\alpha _{cs}'(0)=m$. This leads to highly accurate reduced equation for ${\dot{s}}$ near the stationary point^11^:

34 $$\begin{aligned} {\dot{s}} = -\frac{e_0s}{e_0+ms} \cdot (k_1e_0 - k_{-1}m). \end{aligned}$$

Despite its advantages, this approach has limitations. First, the reduction (34) may be unreliable away from the stationary point, even when $\lambda _- \ll \lambda _+ < 0$ (see Eilertsen et al. 2022b). Second, the anti-funnel construction leads to the following:

### Proposition 1

For (2) with initial condition $(s,c)(0)=(s_0,0)$, let $t=t_{\textrm{cross}}$ be the time the trajectory crosses the upper fence $c=\gamma _c(s)$. Then, the bound

35 $$\begin{aligned} -\frac{k_2e_0s}{K_M+s} \le {\dot{s}} \le -\frac{e_0s}{e_0+ms} \cdot (k_1e_0 - k_{-1}m) \end{aligned}$$

holds for all $t\ge t_{\textrm{cross}}$.

### Proof

Since $\Gamma _{cs}$ is a narrowing anti-funnel, $S_0^{\varepsilon }$ lies between the graphs of $\alpha _{cs}(s)$ and $\gamma _c(s)$. As $S_0^{\varepsilon }$ is invariant, any trajectory entering $\Gamma _{cs}$ cannot cross it. Therefore, $\beta (s) \le c \le \alpha _{cs}(s)$ holds for all $t\ge t_{\textrm{cross}}$ since $\Gamma$ is positively invariant and $\Gamma _{cs} \subset \Gamma$. With ${\dot{s}} = -k_1e_0s + k_1(K_S+s)c$, it follows that

$$\begin{aligned} -k_1e_0s + k_1(K_S+s)\gamma _c(s) \le {\dot{s}} \le -k_1e_0s + k_1(K_S+s)\alpha _{cs}(s) \end{aligned}$$

proving the assertion. $\square$

While the upper bound in (35) is sharper than ${\dot{s}} \le 0$, extracting quantitative information about the sQSSA’s accuracy and dimensionless parameters like $\varepsilon _{RS} = e_0/K_M$ from (35) is not straightforward.

The goal is to define an anti-funnel with fences that provide both qualitative and quantitative information about $S_0^{\varepsilon }$ and the error for a given QSSA. By “trapping” $S_0^{\varepsilon }$ between suitable upper and lower fences that form narrowing anti-funnels, we can derive qualifiers that determine the accuracy and predominance of various QSSAs.

## Trapping the Slow Manifold: The Standard QSSA

As stated in Sect. 3, our strategy is to construct upper and lower fences that form a narrowing anti-funnel. This raises the question: How do we construct suitable fences? One approach is approximating the slow manifold, $S_0^{\varepsilon }$, via perturbation expansion

36 $$\begin{aligned} S_0^{\varepsilon }= h_0(s)+\varepsilon h_1(s) + \varepsilon ^2h_2(s) + {\mathcal {O}}(\varepsilon ^2). \end{aligned}$$

Insertion of (36) into the invariance equation results in a *regular* perturbation problem. However, the coefficients in (36) depend on $\varepsilon$, which in turn depends on a specific TFPV. Since we aim for general error bounds, we need fences that are not heavily $\varepsilon$-dependent.

An alternative approach involves combining quantitative reasoning with creative insights. Kumar and Josić (2011) sought to approximate the flow on $S_0^{\varepsilon }$ for small $k_2$ ($k_2 \mapsto \varepsilon \widehat{k}_2$). They reasoned that

37 $$\begin{aligned} c=\frac{e_0s}{K_M+s} \end{aligned}$$

is a good approximation to $S_0^{\varepsilon }$ when $k_2$ is small. Under this assumption, the flow of *c* on $S_0^{\varepsilon }$ is determined by:

38 $$\begin{aligned} {\dot{c}} \approx \frac{\textrm{d}}{\textrm{d}s}\left( \frac{e_0s}{K_M+s}\right) {\dot{s}} = \frac{K_Me_0}{(K_M+s)^2}\cdot {\dot{s}}. \end{aligned}$$

Substituting (38) and (37) into the conservation law, ${\dot{s}} + {\dot{c}} + k_2c=0$, and solving for ${\dot{s}}$ yields:

39 $$\begin{aligned} {\dot{s}} = -\frac{k_2e_0s(K_M+s)}{K_Me_0 + (K_M+s)^2}. \end{aligned}$$

This reduction is significant for two reasons. First, it effectively captures the Fenichel reduction for this scenario (compare to (19) with small $k_2$). Second, the graph of $\gamma (s)$, where

40 $$\begin{aligned} \gamma : s\mapsto \frac{e_0s}{K_S+s} - \frac{k_2e_0s(K_M+s)}{k_1(K_S+s)(K_Me_0 + (K_M+s)^2)}, \end{aligned}$$

is an excellent candidate for an appropriate anti-funnel fence.

**Fig. 3:**
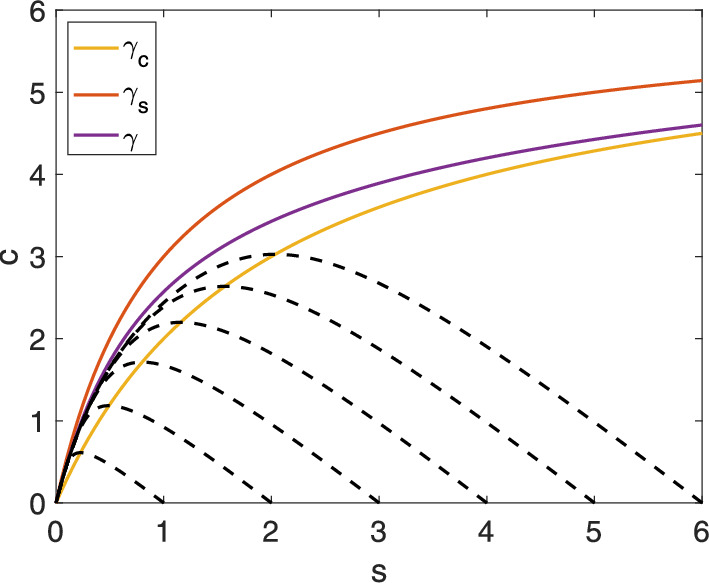
The region between the horizontal nullcline curve $\gamma _c(s)$ and the curve $\gamma (s)$ is a trapping region on the phase plane. The numerical solutions to the mass action equations (1) for several initial conditions are denoted by black dashed lines. $s_0 \in \{1,2,3,4,5,6\}$, $c_0 = 0$, $e_0 = 6$, $k_1 = 1$, $k_{-1} = 1$, $k_2 = 1$

As shown in Fig. 3, $\gamma _c, \gamma _s$ and $\gamma$ are strictly increasing, with $\gamma _c< \gamma < \gamma _s$ for all $s > 0$. The set contained between the graphs of $\gamma _c$ and $\gamma$ is positively invariant:

41 $$\begin{aligned} \Gamma _0:= \{(s,c)\in \mathbb {R}^2_{\ge 0} \;: \; \gamma _c(s) \le c \le \gamma (s)\}. \end{aligned}$$

Let $t_{\textrm{cross}}$ be the time a trajectory crosses the *c*-nullcline, as established in Noethen and Walcher (2007) and Calder and Siegel (2008). Then, $(s,c)(t) \in \Gamma _0$ for all $t \ge t_{\textrm{cross}}$. Importantly:

### Theorem 2

For the differential equation (25), $\Gamma _0$ is a positively invariant, narrowing anti-funnel containing a unique slow manifold, $S_0^{\varepsilon }$.

### Proof

To prove $\Gamma _0$ is positively invariant, we show that the vector field for (2) points inwards at the boundary curves:

42a $$\begin{aligned}&{\dot{c}}(s,\gamma (s)) \;- \gamma '(s){\dot{s}}(s,\gamma (s)) \;< 0 , \end{aligned}$$

42b $$\begin{aligned}&{\dot{c}}(s,\gamma _c(s)) - \gamma _c'(s){\dot{s}}(s,\gamma _c(s)) > 0 . \end{aligned}$$

See “Appendix B” for details. Positive invariance implies trajectories entering $\Gamma _0$ remain within. Calder and Siegel (2008), as well as Noethen and Walcher (2007), prove that for solutions initializing on the *s*-axis, there exists a $t_{\textrm{cross}} > 0$ at which they enter $\Gamma _0$ by crossing $\gamma _c$.

From (42), $\gamma$ and $\gamma _c$ are strong lower and upper fences, respectively, with $\gamma (s) > \gamma _c(s)$ for $s > 0$. Furthermore,

43 $$\begin{aligned} \lim \limits _{s \rightarrow \infty } |\gamma (s) - \gamma _c(s)| = 0 \quad \text {and} \quad \frac{\partial f}{\partial c} (c,s) \ge 0 \end{aligned}$$

in $\Gamma _0$. The assertion follows from Theorem  1. $\square$

Theorem 2 establishes that $\Gamma _0$ contains the slow manifold $S_0^{\varepsilon }$, independent of any specific perturbation scenario. This allows us to extract qualitative and quantitative information about the accuracy of a QSSA and assess predominance when multiple TFPVs are close.

The rest of this section is organized as follows. In Sect. 4.1, we use the improved trapping region to derive a quantitative error estimate for the sQSS approximation and recover the Reich–Sel’kov condition. In Sect. 4.2, we explore the conditions where both the sQSS and reverse QSS reductions are valid, examining their validity as we move away from the Reich–Sel’kov condition.

### Accuracy of the Standard QSSA

To leverage $\Gamma _0$’s properties, we introduce sharp bounds on the substrate depletion for $t \ge t_{\textrm{cross}}$, representing the slow regime.

#### Proposition 2

For system (2) with initial condition $(s, c)(0) = (s_0, 0)$, the bound

44 $$\begin{aligned} -\frac{k_2e_0s}{K_M + s} \le {\dot{s}} \le -\frac{k_2e_0s(K_M+s)}{K_Me_0 + (K_M+s)^2} \end{aligned}$$

holds for all $t \ge t_{\textrm{cross}}$.

#### Proof

The proof follows from the construction of $\Gamma _0$ and substituting $\gamma _c(s) \le c \le \gamma (s)$ into (2a). $\square$

Since the LHS of (44) is the sQSSA for substrate depletion, the design of $\Gamma _0$ suggests that the sQSSA performs well when $\gamma$ and $\gamma _c$ are close. Rewriting (44) as:

45 $$\begin{aligned} -\frac{k_2e_0s}{K_M+s} \le {\dot{s}} \le -\frac{k_2e_0s}{K_M+s}\left( \frac{1}{1 + \varphi (s)}\right) , \quad \varphi (s) = \frac{K_Me_0}{(K_M+s)^2} \end{aligned}$$

we see that, for any subinterval $\ell \subset [0,s_0]$,

46 $$\begin{aligned} -\frac{k_2e_0s}{K_M+s} \le {\dot{s}} \le -\frac{k_2e_0s}{K_M+s}\left( \frac{1}{1 + \displaystyle \max _{s\in \ell }\varphi (s)}\right) ,\quad t_{\textrm{cross}} \le t. \end{aligned}$$

Since

47 $$\begin{aligned} \displaystyle \max _{0\le s \le s_0}\varphi (s) = \frac{e_0}{K_M} = \varepsilon _{RS}, \end{aligned}$$

we have:

48 $$\begin{aligned} -\frac{k_2e_0s}{K_M+s} \le {\dot{s}} \le -\frac{k_2e_0s}{K_M+s}\cdot \sum _{j=0}^{\infty }(-1)^j\varepsilon _{RS}^j, \quad t_{\textrm{cross}}\le t, \quad \varepsilon _{RS} < 1. \end{aligned}$$

This analysis has several significant consequences. First, we have established the sQSSA’s asymptotic dependency on $\varepsilon _{RS}$ without relying on non-dimensionalization, unlike traditional methods (see Heineken et al. 1967; Reich and Sel’kov 1974; Segel and Slemrod 1989, among others). This is advantageous due to the non-uniqueness of non-dimensionalization, which can lead to different “small parameters” (Heineken et al. 1967; Reich and Sel’kov 1974; Segel 1988). Second, the bound (48) is more aesthetic than the estimate in Eilertsen et al. (2024a):

49 $$\begin{aligned} -\frac{k_2e_0s}{K_M+s} \le {\dot{s}} \le -(1-m)\frac{k_2e_0s}{K_M+s}, \quad t_{\textrm{cross}}\le t, \end{aligned}$$

where “*m*" is the slope of the slow tangent vector, $v^+$.

Third, and this is somewhat unexpected, we gain more information about the approximation error of the flow on $S_0^{\varepsilon }$ from $\varphi (s)$ than from *m*. From (45), the sQSSA approximates the flow on $S_0^{\varepsilon }$ well in the regions where:

50 $$\begin{aligned} K_Me_0 \ll (K_M+s)^2, \end{aligned}$$

even if $\varepsilon _{RS} \sim 1$. In particular, the sQSSA is a good approximation in regions where:

51 $$\begin{aligned} \max \{K_M,e_0\} \ll s. \end{aligned}$$

This relates to the validity of the reverse quasi-steady-state approximation (rQSSA), which operates in high enzyme concentrations. To revisit the sQSSA’s predominance, we examine the rQSSA’s validity and investigate any overlap in their conditions.

### Validity of the sQSSA for High Enzyme Concentrations

Equation (45) implies that the sQSSA is an excellent approximation to the flow on $S_0^{\varepsilon }$ when $K_M \ll s$ and $e_0 \lesssim s$. In this region, the graphs of the *c*-nullcline and $\gamma (s)$ approach their horizontal asymptote, $c=e_0$, reflected in $\varphi (s) \rightarrow 0$ as $s\rightarrow \infty$. The slow manifold, $S_0^{\varepsilon }$, is nearly horizontal implying ${\dot{c}} \approx 0$ on $S_0^{\varepsilon }$ even for larger $\varepsilon _{RS}$.

Two singular perturbation scenarios lead to a nearly horizontal slow manifold: the sQSSA, where the slow manifold coalesces with the *s*-axis as $k_1e_0 \rightarrow 0$, and the rQSSA, coinciding with small $k_{-1}$ and small $k_{2}$:

52a $$\begin{aligned} {\dot{s}}&= -k_1(e_0-c)s+\varepsilon \widehat{k}_{-1}c, \end{aligned}$$

52b $$\begin{aligned} {\dot{c}}&= \;\;\;k_1(e_0-c)s -\varepsilon \widehat{k}_{-1} -\varepsilon \widehat{k}_2. \end{aligned}$$

In the rQSSA scenario, the set of stationary points in the singular limit is not a submanifold of $\mathbb {R}^2$:

$$\begin{aligned} S_0 = S_0^{(1)} \cup S_0^{(2)} \end{aligned}$$

where^12^

53a $$ \begin{aligned} S_0^{(1)}&= \{(s,c)\in \mathbb {R}^2_{\ge 0}: s=0\; \& \;0 \le c\le e_0-\zeta _1\}, \quad 0< \zeta _1 < e_0, \end{aligned}$$

53b $$ \begin{aligned} S_0^{(2)}&= \{(s,c)\in \mathbb {R}^2_{\ge 0}: c=e_0\; \& \;\zeta _2 \le s \le s_0\}, \;\qquad 0< \zeta _2 < s_0. \end{aligned}$$

Classical Fenichel theory does not apply to the entire set, but it applies to specific compact submanifolds. The resulting Fenichel reduction via projection onto $TS_0^{(2)}$ is:

54a $$\begin{aligned} {\dot{s}}&= -k_2e_0, \end{aligned}$$

54b $$\begin{aligned} {\dot{c}}&= 0. \end{aligned}$$

Likewise, the Fenichel reduction obtained via projection onto $TS_0^{(1)}$ is:

55a $$\begin{aligned} {\dot{s}}&= 0, \end{aligned}$$

55b $$\begin{aligned} {\dot{c}}&= -k_2c. \end{aligned}$$

In the rQSSA, trajectories initially approach the line $c=e_0$ and stay close until reaching the vicinity of the transcritical bifurcation point, $(s,c)=(0,e_0)$. Near this point, trajectories approach (0, 0) as $t\rightarrow \infty$; however, the slow eigenvector in this scenario is nearly indistinguishable from the *c*-axis (and in fact aligns with the *c*-axis in the singular limit).

The rQSSA’s long-time validity requires $\varepsilon _{RS}^{-1} \ll 1$, while the sQSSA’s long-time validity requires $\varepsilon _{RS} \ll 1$ (Eilertsen and Schnell 2020). However, through comparison, the sQSSA with (54) reveals that they are practically indistinguishable when $K_M \ll e_0 \lesssim s$. Thus, the sQSSA can approximate the flow on $S_0^{\varepsilon }$ to the right of the bifurcation point, extending its validity beyond the Reich–Sel’kov parameter.

A natural question is: If we use the sQSSA to approximate the flow on $S_0^{\varepsilon }$ to the right of the bifurcation instead of (54), how reliable is it in an asymptotic sense? Since $S_0^{\varepsilon }$ lies within $\Gamma _0$ when $0<\varepsilon$, we can get a rough answer. Assuming $\varepsilon _{RS}^{-1} \ll 1$ and considering $s\in [e_0,s_0]$, it follows from (45) that, for $t\ge t_{\textrm{cross}}$,

56 $$\begin{aligned} \begin{aligned} -\frac{k_2e_0s}{K_M+s} \le {\dot{s}}&\le -\frac{k_2e_0s}{K_M+s}\left( \frac{1}{1+\displaystyle \max _{s\in [e_0,s_0]}\varphi (s)}\right) \\&= -\frac{k_2e_0s}{K_M+s}\left( \frac{1}{1+\displaystyle \mu }\right) , \quad \mu :=\frac{\varepsilon _{RS}}{(1+\varepsilon _{RS})^2}< \varepsilon _{RS}^{-1}\\&\le -\frac{k_2e_0s}{K_M+s}\left( \frac{1}{1+\varepsilon _{RS}^{-1}}\right) \\&=-\frac{k_2e_0s}{K_M+s}\cdot \sum _{j=0}^{\infty }(-1)^j\varepsilon _{RS}^{-j}, \quad 1<\varepsilon _{RS}. \end{aligned} \end{aligned}$$

The bound (56) implies the sQSSA is a good approximation when $\varepsilon _{RS}^{-1}\ll 1$ and $e_0 \lesssim s$, improving as $e_0/s \rightarrow 0$.

While the sQSSA approximates the flow on $S_0^{\varepsilon }$ well when $\varepsilon _{RS}^{-1} \ll 1$ and $e_0\lesssim s$, it cannot be equipped with $s_0$ as the initial substrate concentration in the rQSSA scenario. If the initial condition is $(s,c)(0)=(s_0,0)$, with $e_0 < s_0$, Fenichel theory dictates that the reduction (54) should be equipped with $(s,c)=(s_0-e_0,e_0).$ This extends to the sQSSA if used to approximate the flow on $S_0^{\varepsilon }$ to the right of the bifurcation point when $\varepsilon _{RS}^{-1}\ll 1$.

The existence of multiple accurate QSS reductions under the same conditions necessitates evaluating which QSSA is predominant. To truly verify the standard-QSS reduction’s applicability, we must explore other QSS reductions near the sQSS curve in the phase-plane. As noted in Sect. 3, in scenarios like large $k_{-1}$, the sQSSA (17) and the slow product QSSA (19) are indistinguishable. To find the most accurate reduced system and its validity conditions, we next investigate the slow manifold’s location relative to the (algebraic) variety that generates the slow product QSS reduction (19).

## Trapping the Slow Manifold: The Slow Product QSSA

This section defines a new upper fence and anti-funnel for the slow manifold using the slow product QSS curve under specific parametric restrictions. We compare this to previous results to assess the approximation accuracy of the sQSSA (17) and the slow product QSSA (19), revisiting the Reich–Sel’kov condition and investigating whether it ensures the predominance of the sQSSA.

**Fig. 4:**
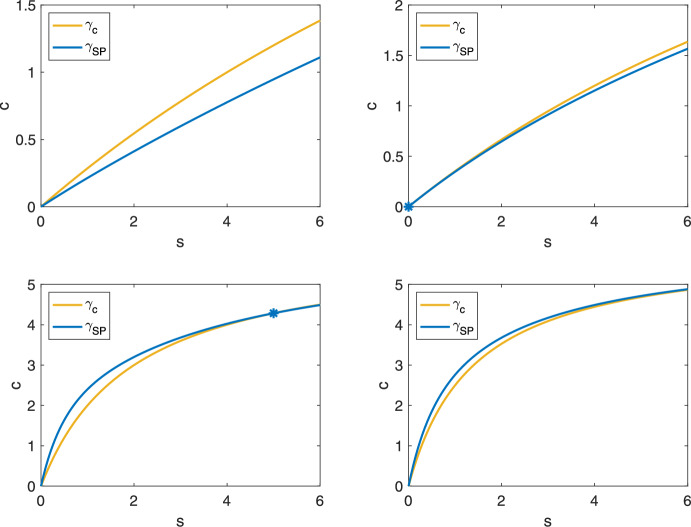
The slow product QSS curve $\gamma _{SP}$ lies above the horizontal nullcline $\gamma _c$ for $s < s^*$ (58) in the phase plane. Top Left ($k_2 > e_0k_1$): $k_1 = 0.1$, $k_2 = 1$, $k_{-1} = 1$, $e_0 = 6$, $s^* = -4$. Top Right ($k_2 = e_0k_1$): $k_1 = 0.1$, $k_2 = 0.6$, $k_{-1} = 1$, $e_0 = 6$, $s^* = 0$. Bottom Left ($k_2 < e_0k_1$): $k_1 = 1$, $k_2 = 1$, $k_{-1} = 1$, $e_0 = 6$, $s^* = 5$. Bottom Right ($k_2 < e_0k_1$): $k_1 = 1$, $k_2 = 0.4$, $k_{-1} = 1$, $e_0 = 6$, $s^* = 14$

The slow product QSS reduction (19), derived from Fenichel theory for small $k_2$, corresponds to the QSS curve

57 $$\begin{aligned} c = \gamma _{SP}(s):= \frac{e_0s}{K_S+s} - \frac{k_2e_0s}{k_1(K_Se_0 + (K_S+s)^2)}. \end{aligned}$$

This closely resembles the curve $\gamma (s)$. In Sect. 4.1, we established that the slow manifold lies between $\gamma (s)$ and $\gamma _c(s)$. Now, we explore how the slow product QSS variety fits into these findings and the insights its location provides concerning the sQSSA’s validity and predominance.

In the phase plane, $\gamma _{SP}(s)$ lies above the horizontal nullcline $\gamma _c(s)$ for $s < s^*$ where

58 $$\begin{aligned} s^* = \frac{k_{-1}e_0}{k_2} - \frac{k_{-1}}{k_1} = \frac{k_{-1}}{k_2}(e_0 - K) \end{aligned}$$

is their intersection point for substrate concentration. We are primarily interested in cases where $s^*$ is positive, placing the intersection within the first quadrant. When $s^*$ is negative, or equivalently, when

59 $$\begin{aligned} e_0 < K, \end{aligned}$$

the slow manifold crosses the sQSS variety, and the sQSSA is the best known reduction.

However, as $e_0$ increases, $s^*$ shifts to the right. Figure 4 shows how the curves’ positions change with parameter values. When $e_0 > K$, the intersection point lies in the first quadrant and $\gamma _{SP}(s) \ge \gamma _c(s)$ for $0 \le s \le s^*$. Also, $\gamma _{SP}(s) \le \gamma (s)$ for all $s \ge 0$. Thus, the slow product QSS curve lies within $\Gamma _0$ for a significant portion of the phase plane. This raises the questions: Do solutions cross $\gamma _{SP}(s)$ after crossing $\gamma _c(s)$ under any parametric conditions? If so, can we locate the slow manifold more precisely?

**Fig. 5:**
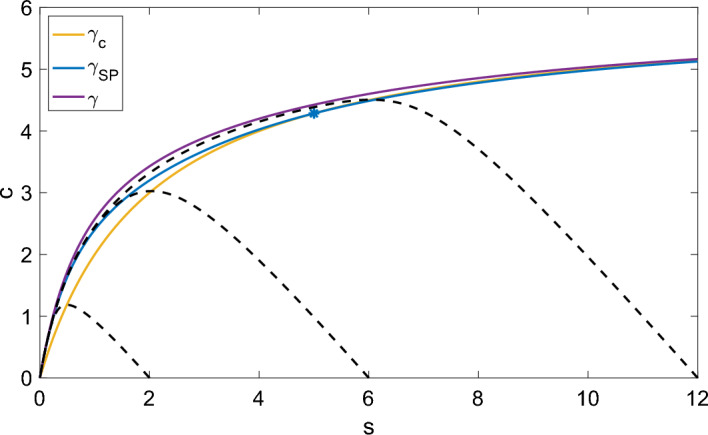
The region between the slow product QSS curve $\gamma _{SP}(s)$ and the curve $\gamma (s)$ is a trapping region for solutions in the phase plane when $e_0 < 8K_S$. The intersection point of $\gamma _{SP}$ and $\gamma _c$, $s^*$, is denoted by the blue star. The numerical solutions to the mass action equations (1) for several initial conditions are denoted by black dashed lines. $s_0 \in \{2,6,12\}$, $c_0 = 0$, $e_0 = 6$, $k_1 = 1$, $k_{-1} = 1$, $k_2 = 1$

Numerical evidence suggests this is possible, with solutions lying closer to the slow product QSS curve in the steady-state regime (see Fig. 5). Interestingly, the parametric conditions for this also ensure the slow-product QSS reduction’s dominance over the sQSS reduction, aligning with our overall goal.

Our main results establish the conditions for positive invariance of the set bordered by the graphs of $\gamma _{SP}(s)$ and $\gamma (s)$:

60 $$\begin{aligned} \Gamma _{SP}:= \left\{ (s,c) \in \mathbb {R}^2_{\ge 0}\;: \; \;\gamma _{SP}(s) \le c \le \gamma (s)\right\} , \end{aligned}$$

and show that all solutions eventually cross $\gamma _{SP}(s)$ under those conditions.

### Theorem 3

For a solution (*s*, *c*)(*t*) to (2) with initial conditions $(s,c)(0) = (s_0,0)$. If $e_0 < 8K_S$, then there exists a $t_{\mathrm{cross\_sp}} > 0$ at which the trajectory crosses $\gamma _{SP}(s)$, and

61 $$\begin{aligned} (s,c)(t) \in \Gamma _{SP}, \quad \forall \; t \ge t_{\mathrm{cross\_sp}}. \end{aligned}$$

### Proof

To establish positive invariance, we show that the vector field at the boundary curve $\gamma _{SP}$ points towards $\Gamma _{SP}$ when $e_0 < 8K_S$:

62 $$\begin{aligned} {\dot{c}}(s,\gamma _{SP}(s)) - \gamma _{SP}'(s){\dot{s}}(s,\gamma _{SP}(s)) > 0 \end{aligned}$$

See “Appendix C’ for details. Combining this with (42a) ensures solutions entering $\Gamma _{SP}$ remain within.

We also show that solutions beginning outside $\Gamma _{SP}$ eventually enter it. In contradiction, assume that a solution starting at $(s_0,0)$ converges to the stationary point (0, 0) without entering $\Gamma _{SP}$. Then, the slope of the tangent to $c = \gamma _{SP}(s)$ at $s = 0$ should be greater than *m* (31). However, we show that

63 $$\begin{aligned} \gamma _{SP}'(0) < m \end{aligned}$$

always holds, where:

64 $$\begin{aligned} \gamma _{SP}'(0) = \frac{k_1e_0}{k_{-1}} - \frac{k_2e_0}{k_{-1}(K_S+e_0)}. \end{aligned}$$

When $e_0 < K$, $\gamma '_{SP}(0) < \gamma '_c(0)$ holds, and Theorem 2 implies $\gamma '_c(0) < m$, demonstrating $\gamma '_{SP}(0) < m$. For $e_0 > K$, see “Appendix D” for proof of (63). Thus, our assumption is false, and a $t_{\mathrm{cross\_sp}} > 0$ exists when the trajectory crosses $\gamma _{SP}(s)$. The proof is complete by combining the two arguments. $\square$

The primary benefit of establishing the positive invariance of $\Gamma _{SP}$ is that it allows for a more precise localization of the slow manifold.

### Theorem 4

If $e_0 < 8K_S$, then for the differential equation (25), $\Gamma _{SP}$ is a positively invariant, narrowing anti-funnel within which there exists a unique slow manifold, $S_0^{\varepsilon }$.

### Proof

Inequality (62) ensures $c = \gamma _{SP}(s)$ is a strong upper fence for (25) when $e_0 < 8K_S$. It is verifiable that $\gamma (s) > \gamma _{SP}(s)$ for $s > 0$ and:

65 $$\begin{aligned} \lim \limits _{s \rightarrow \infty } |\gamma (s) - \gamma _{SP}(s)| = 0 \end{aligned}$$

implying $\Gamma _{SP}$ (41) is a narrowing anti-funnel for $e_0 < 8K_S$. Further,

66 $$\begin{aligned} \frac{\partial f}{\partial c} (c,s) \ge 0 \end{aligned}$$

in $\Gamma _{SP}$, and the claim follows from Theorem 1. $\square$

**Fig. 6:**
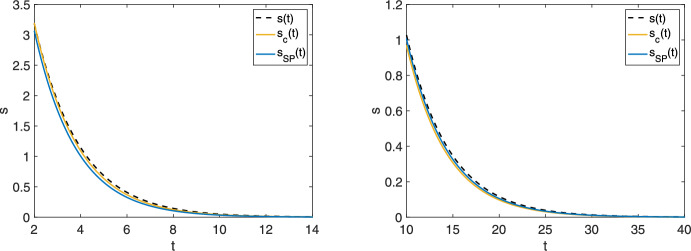
The condition $e_0 < K$ ensures that the sQSSA provides a better approximation than the slow product QSSA under the Reich–Sel’kov condition. $e_0 \ll K_M$ is satisfied in both panels. Left ($e_0 < K$): $s_0 = 9$, $c_0 = 0$, $e_0 = 6$, $k_1 = 1$, $k_{-1} = 100$, $k_2 = 10.$ Right ($e_0 > K$): $s_0 = 9$, $c_0 = 0$, $e_0 = 6$, $k_1 = 1$, $k_{-1} = 100$, $k_2 = 4$. Substrate depletion over time is shown in both panels. The numerical solution to the mass action equations (1) is denoted by black dashed lines. $s_c(t)$ is the numerical solution to (17) and $s_{SP}(t)$ is the numerical solution to (19)

The slow manifold is contained within $\Gamma _{SP}$ when $e_0 < 8K_S$. While this condition might seem restrictive, it connects directly to the sQSSA’s validity analysis. We show that the slow manifold lies closer to the slow product QSS curve than the sQSS curve when $e_0 < 8K_S$. The remaining task is to analyze the implications of this location.

### Revisiting the Reich–Sel’kov Condition

The Reich–Sel’kov qualifier ensures the sQSS reduction’s accuracy, as validated in Sect. 4 using the novel anti-funnel (2). Our analysis shows that the sQSSA is the best known approximation to the slow manifold under most parametric scenarios. For instance, recall the intersection point (58). When $e_0 < K$, the intersection is inconsequential. Consequently, when the Reich–Sel’kov condition $e_0 \ll K_M$ is combined with $e_0 < K$, the sQSSA is the predominant approximation.

What happens when $e_0 \ll K_M$, $e_0 \ll K_S$ (ensuring $e_0 < 8K_S$), but $e_0 > K$? Theorem 3 implies that trajectories lie closer to $\gamma _{SP}$ than to $\gamma _c$ for a significant portion of the slow dynamics (see Fig. 5), and therefore

67 $$\begin{aligned} {-\frac{k_2e_0s}{K_M+s} \le -\frac{k_2e_0s(K_S+s)}{K_Se_0+(K_S+s)^2} \le {\dot{s}} \le -\frac{k_2e_0s(K_M+s)}{K_Me_0+(K_M+s)^2}} \end{aligned}$$

after the trajectories cross $\gamma _{SP}$ and $s \le s^*$. Moreover, we know that the slope *m* of the linear approximation is greater than the slope of the tangent to $\gamma _{SP}$ at the origin (63) and $\gamma _{SP}'(0) > \gamma _c'(0)$ whenever $e_0 > K$. Thus, the approximation errors near the stationary point follow:

68 $$\begin{aligned} m - \gamma _{SP}'(0) < m - \gamma _c'(0) \end{aligned}$$

when $e_0 > K$. Consequently, complementing the Reich–Sel’kov condition with the stronger condition $e_0 < K$ is necessary to guarantee that the sQSS is indeed the predominant quasi-steady-state approximation of the MM reaction mechanism, and this may be relevant in the context of the inverse problem where parameters are estimated at low substrate concentration (Stroberg and Schnell 2016): If $e_0 > K$, using the slow product QSS variety as an approximation for small *s* is beneficial. Figure 6 illustrates the numerical substrate depletion curves. The restrictive condition $e_0 < K$ is necessary to ensure the sQSS most accurately approximates the MM dynamics for small substrate concentrations.

## Discussion

This work addresses a critical gap in the understanding of the quasi-steady-state approximation for the MM reaction mechanism. While the sQSSA is a widely used tool in enzyme kinetics, the precise conditions for its validity have remained a topic of ongoing investigation. We conducted a thorough phase-plane analysis of the MM mass-action kinetics using the theory of fences and anti-funnels, a powerful technique for analyzing the long-time behavior of dynamical systems.

Our analysis led to the identification of new positively invariant sets that contain the slow manifold, $S_0^{\varepsilon }$, in the substrate-complex phase plane. These sets provide valuable insights into the dynamics of the system and allow for a more precise characterization of the sQSSA’s accuracy. As a result, we obtained improved bounds on the estimation error of various QSS approximations, including the standard, reverse, and slow product formation approximations, in the slow regime.

Significantly, we have demonstrated that the commonly accepted qualifier for the validity of the sQSSA, the Reich–Sel’kov condition ($e_0 \ll K_M$), does not guarantee that the sQSSA is the predominant reduction. Predominance necessitates a more restrictive condition

69 $$\begin{aligned} e_0 \ll K, \end{aligned}$$

where *K* is the Van Slyke-Cullen constant. This finding challenges the traditional understanding of the sQSSA’s validity and highlights the importance of considering both accuracy and predominance when evaluating QSS approximations.

It is important to note that our analysis primarily focused on the approximation error in the slow regime, specifically the error between the Fenichel reduction and the actual flow on the slow manifold, $S_0^{\varepsilon }$. However, two primary sources of error contribute to the overall accuracy of approximate solutions to singularly perturbed ODEs. The first is the error in approximating the flow on the slow manifold itself, as addressed in our analysis. The second is the error in approximating the trajectory’s approach to the slow manifold, including timescale estimates such as $t_{\textrm{cross}}$, which demarcates the intersection of trajectories with pertinent QSS varieties. This latter source of error is generally more challenging to analyze. Although some progress has been made in this area (see Eilertsen et al. 2024a), further exploration and improvement is required.

Our findings have broader implications for the application of QSS approximations in various fields. By providing a more refined understanding of the sQSSA’s validity, our work can guide researchers in selecting the most appropriate and accurate reduction for their specific needs. This is particularly crucial in areas such as quantitative biology and pharmacology, where accurate model reduction is essential for understanding complex biological processes and designing effective therapeutic interventions.

Future research could extend our analysis by considering more complex reaction mechanisms or incorporating additional factors that might influence the sQSSA’s validity. Further investigation of the error associated with the trajectory’s approach to the slow manifold is also warranted. By addressing these open questions, we can continue to refine our understanding of QSS approximations and enhance their utility in diverse scientific disciplines.

## Data Availability

We do not analyse or generate any datasets, because our work proceeds within a theoretical and mathematical approach.

## Appendices

These appendices provide supplementary information to enhance the understanding of the main text. The appendices include a synopsis of the theory of fences, funnels, and anti-funnels, as well as detailed calculations and proofs for key results presented in the paper.

## Appendix A: Synopsis of the Theory of Fences, Funnels and Antifunnels

This section highlights the notation of fences and anti-funnels within the context of the Michaelis-Menten system.

In general, an anti-funnel is the region above an upper fence and below a lower fence in a two-dimensional phase space. Typically, anti-funnels (or funnels) are visualized with the independent variable increasing from left to right. The vector field points outwards at the fences of an anti-funnel, while it points inwards at the fences of a funnel. Consequently, solutions generally leave anti-funnels. The anti-funnel theory implies that all but one solution eventually leave a narrowing anti-funnel (see Hubbard and West 1995, Chapter1). This property makes fences and anti-funnels useful for identifying a unique, exceptional solution that remains within the anti-funnel.

In the context of our work on the Michaelis-Menten system, the substrate concentration, *s*, decreases over time, with $s(0) = s_0$ and $s(t) \rightarrow 0$ as $t \rightarrow \infty$. In the (*s*, *c*) phase-plane, solution trajectories start at the substrate axis and move from right to left with respect to the *s*-axis. Thus, the vector field always has a negative component in the *s*-direction.

Despite this difference in directionality, the concept of anti-funnels remains applicable. The vector field can still point inwards at the anti-funnel fences in the (*s*, *c*) coordinate system as we move from right to left, while satisfying the conditions in Definition 1. Consequently, solution trajectories can enter the anti-funnels defined in the paper.

It is important to remember the nomenclature of upper and lower fences in this context. The slow manifold lies above the upper fence and below the lower fence in the phase plane.

## Appendix B: $\Gamma _0$ is a Positively Invariant Set for Solutions in the Phase Plane

This section provides the detailed calculations to prove inequality (42a), which establishes the positive invariance of the set $\Gamma _0$. We begin by evaluating the dynamics at the curve $c = \gamma (s)$:

B.1a $$\begin{aligned} {\dot{s}}(s,\gamma (s))&= -k_1e_0s + k_1(K_S+s)\gamma (s), \end{aligned}$$

B.1b $$\begin{aligned}&= -k_1e_0s + k_1(K_S+s)\left( \frac{e_0s}{K_S+s} - \frac{k_2e_0s(K_M+s)}{k_1(K_S+s)(K_Me_0 + (K_M+s)^2)}\right) , \end{aligned}$$

B.1c $$\begin{aligned}&= -\frac{k_2e_0s(K_M+s)}{K_Me_0 + (K_M+s)^2}, \end{aligned}$$

and

B.2a $$\begin{aligned} {\dot{c}}(s,\gamma (s))&= k_1e_0s - k_1(K_M+s)\gamma (s), \end{aligned}$$

B.2b $$\begin{aligned}&= k_1e_0s - k_1(K_M+s)\left( \frac{e_0s}{K_S+s} - \frac{k_2e_0s(K_M+s)}{k_1(K_S+s)(K_Me_0 + (K_M+s)^2)}\right) , \end{aligned}$$

B.2c $$\begin{aligned}&= -\frac{k_2e_0s}{K_S+s} + \frac{k_2e_0s(K_M+s)^2}{(K_S+s)(K_Me_0 + (K_M+s)^2)}. \end{aligned}$$

The derivative of $\gamma (s)$ is:

B.3 $$\begin{aligned} \gamma '(s)&= \frac{K_S e_0}{(K_S+s)^2} - \frac{k_2K_Se_0(K_M+s)}{k_1(K_S+s)^2(K_Me_0 + (K_M+s)^2)} \end{aligned}$$

B.4 $$\begin{aligned}&\quad - \frac{k_2e_0s}{k_1(K_S+s)(K_Me_0 + (K_M+s)^2)} + \frac{2k_2e_0s(K_M+s)^2}{k_1(K_S+s)(K_Me_0 + (K_M+s)^2)^2}. \end{aligned}$$

The required expression at $c = \gamma (s)$ is:

B.5a $$\begin{aligned} {\dot{c}} - \gamma '(s){\dot{s}}&= -\frac{k_2e_0s}{K_S+s} + \frac{k_2e_0s(K_M+s)^2}{(K_S+s)(K_Me_0 + (K_M+s)^2)} + \frac{k_2e_0s(K_M+s)}{K_Me_0 + (K_M+s)^2} \gamma '(s), \end{aligned}$$

B.5b $$\begin{aligned}&= -\frac{k_2e_0s}{(K_S+s)(K_Me_0 + (K_M+s)^2)}\left( K_Me_0 - (K_M+s)(K_S+s)\gamma '(s)\right) . \end{aligned}$$

To prove inequality (42a), we need to show that $\delta (s) = K_Me_0 - (K_M+s)(K_S+s)\gamma '(s) > 0$. This also proves that $\gamma (s)$ is a lower fence. Substituting $\gamma '(s)$ and simplifying, we obtain:

$$\begin{aligned} \delta (s)&= K_Me_0 - \frac{K_Se_0(K_M+s)}{(K_S+s)} + \frac{k_2K_Se_0(K_M+s)^2}{k_1(K_S+s)(K_Me_0 + (K_M+s)^2)} \\&\quad + \frac{k_2e_0s(K_M+s)}{k_1(K_Me_0 + (K_M+s)^2)} - \frac{2k_2e_0s(K_M+s)^3}{k_1(K_Me_0+(K_M+s)^2)^2}, \\&= \frac{k_2e_0}{k_1(K_S+s)(K_Me_0 + (K_M+s)^2)^2}\Bigl ((K_Me_0 + (K_M+s)^2 s)^2 \\&\qquad + K_S(K_M+s)^2(K_Me_0 + (K_M+s)^2) \\ &\qquad \qquad + (K_M+s)(K_S+s)(K_Me_0 + (K_M+s)^2)s - 2(K_M+s)^3(K_S+s)s\Bigl ). \end{aligned}$$

Expanding the first term in the expression,

$$\begin{aligned} \delta (s)&= \frac{k_2e_0}{k_1(K_S+s)(K_Me_0 + (K_M+s)^2)^2}\Bigl ((K_Me_0)^2s + 2(K_Me_0)(K_M+s)^2s \\&\qquad + (K_M+s)^4s + K_S(K_M+s)^2(K_Me_0 + (K_M+s)^2) \\ &\qquad \qquad + (K_M+s)(K_S+s)(K_Me_0 + (K_M+s)^2)s - 2(K_M+s)^3(K_S+s)s\Bigl ), \\&= \frac{k_2e_0}{k_1(K_S+s)(K_Me_0 + (K_M+s)^2)^2}\Bigl ((K_Me_0)^2s + 2(K_Me_0)(K_M+s)^2s \\&\qquad + (k_2/k_1)(K_M+s)^3 s + K_S(K_M+s)^2(K_Me_0 + (K_M+s)^2) \\&\qquad \qquad + K_Me_0(K_M+s)(K_S+s)s\Bigl ), \end{aligned}$$

This expression is a sum of positive terms, proving inequality (42a).

For inequality (42b), a straightforward calculation at $c = \gamma _c(s)$ gives:

$$\begin{aligned} {\dot{c}} - \gamma _c'(s){\dot{s}} = 0 + \left( \frac{K_Me_0}{(K_M+s)^2}\right) \left( \frac{k_2e_0s}{K_M+s}\right) \end{aligned}$$

which is always positive for $s > 0$. Thus, $\Gamma _0$ is a positively invariant region.

## Appendix C: Positive Invariance of $\Gamma _{SP}$ When $e_0 < 8K_S$

This section provides the detailed calculations to prove inequality (62), which establishes the positive invariance of the set $\gamma _{SP}$ when $e_0 < 8K_S$.

We first compute the differential terms at $\gamma _{SP}$:

C.1a $$\begin{aligned} {\dot{c}}(s,\gamma _{SP}(s))&= k_1e_0s - k_1(K_M+s)\gamma _{SP}(s), \end{aligned}$$

C.1b $$\begin{aligned}&= k_1e_0s - k_1e_0s\frac{(K_M+s)}{(K_S+s)} + \frac{k_2e_0s(K_M+s)}{K_Se_0 + (K_S+s)^2}, \end{aligned}$$

C.1c $$\begin{aligned}&= -\frac{k_2e_0s}{(K_S+s)} + \frac{k_2e_0(K_M+s)s}{K_Se_0 + (K_S+s)^2}, \end{aligned}$$

and

C.2a $$\begin{aligned} {\dot{s}}(s,\gamma _{SP}(s))&= -k_1e_0s + k_1(K_S+s)\gamma _{SP}(s), \end{aligned}$$

C.2b $$\begin{aligned}&= -\frac{k_2e_0(K_S+s)s}{K_Se_0 + (K_S+s)^2}. \end{aligned}$$

The expression at $c = \gamma _{SP}(s)$ is:

C.3a $$\begin{aligned} {\dot{c}} - \gamma _{SP}'(s){\dot{s}}&= -\frac{k_2e_0s}{(K_S+s)} + \frac{k_2e_0(K_M+s)s}{K_Se_0 + (K_S+s)^2} + \frac{k_2e_0(K_S+s)s}{K_Se_0 + (K_S+s)^2}\gamma _{SP}'(s), \end{aligned}$$

C.3b $$\begin{aligned}&= \frac{k_2e_0s}{(K_S+s)(K_Se_0 + (K_S+s)^2)} \bigl (-(K_Se_0 + (K_S+s)^2) + \end{aligned}$$

C.3c $$\begin{aligned}&\qquad (K_M+s)(K_S+s) + (K_S+s)^2\gamma _{SP}'(s) \bigr ) \end{aligned}$$

Proving inequality (62) simplifies to showing:

C.4 $$\begin{aligned} -(K_Se_0 + (K_S+s)^2) + (K_M+s)(K_S+s) + (K_S+s)^2\gamma _{SP}'(s) > 0. \end{aligned}$$

This also proves that $\gamma _{SP}(s)$ is an upper fence. Substituting $\gamma _{SP}'(s)$:

C.5a $$\begin{aligned} \gamma _{SP}'(s) = \frac{K_Se_0}{(K_S+s)^2} - \frac{k_2e_0}{k_1(K_Se_0 + (K_S+s)^2)} + \frac{2k_2e_0(K_S+s)s}{k_1(K_Se_0 + (K_S+s)^2)^2}, \end{aligned}$$

and simplifying, we obtain:

C.6 $$\begin{aligned} \frac{k_2}{k_1}(K_S+s) - \frac{k_2e_0(K_S+s)^2}{k_1(K_Se_0 + (K_S+s)^2)} + \frac{2k_2e_0(K_S+s)^3s}{k_1(K_Se_0 + (K_S+s)^2)^2} \end{aligned}$$

which is positive for $e_0 < 8K_S$. To show this, we further simplify the inequality to:

C.7a $$\begin{aligned} (K_S+s)^4 + e_0(K_S+s)^3 - K_Se_0^2s&> 0, \end{aligned}$$

C.7b $$\begin{aligned} 1 + \frac{e_0}{(K_S+s)} - \frac{K_Se_0^2s}{(K_S+s)^4}&> 0. \end{aligned}$$

Substituting $e_0 = \eta K_S$ and introducing $x = K_S/(K_S+s)$, we get:

C.8 $$\begin{aligned} 1 + \frac{e_0}{(K_S+s)} - \frac{K_Se_0^2s}{(K_S+s)^4} = 1 + \eta x - \eta ^2 x^3(1-x) = \eta ^2x^4 - \eta ^2x^3 + \eta x + 1. \nonumber \\ \end{aligned}$$

We want to show that $\eta ^2x^4 - \eta ^2x^3 + \eta x + 1 > 0$ for $\eta < 8$. Dividing by $\eta ^2$ and factoring the quartic function, we get:

C.9 $$\begin{aligned} x^4 - x^3 + \frac{1}{\eta } x + \frac{1}{\eta ^2} = \left( x^2 + \bar{\eta }_+x - \frac{1}{\eta }\right) \left( x^2 + \bar{\eta }_-x - \frac{1}{\eta }\right) \end{aligned}$$

where

$$\begin{aligned} \bar{\eta }_{\pm } = -\frac{1}{2} \pm \frac{1}{2}\sqrt{\frac{\eta - 8}{\eta }}. \end{aligned}$$

This quartic function has no real roots when $\eta < 8$ and remains positive. It has 2 real repeated roots for $\eta = 8$ and 4 real roots when $\eta > 8$. This completes the proof for the first part of Theorem 3.

## Appendix D: Solutions Approach the Origin at a Slope Greater than $\gamma _{SP}'(0)$

This section provides the details to prove the inequality:

D.1 $$\begin{aligned} m - \gamma '_{SP}(0) > 0 \end{aligned}$$

when $k_2 < k_1e_0$, completing the proof for Theorem 3.

Recall:

D.2 $$\begin{aligned} m = \frac{1}{2k_{-1}}\left( -k_{-1} - k_2 + k_1e_0 + \sqrt{(k_{-1} + k_2 + k_1e_0)^2 - 4k_1k_2e_0}\right) \end{aligned}$$

and

D.3 $$\begin{aligned} \gamma _{SP}'(0) = \frac{k_1e_0}{k_{-1}} - \frac{k_2k_1e_0}{k_{-1}(k_1e_0+k_{-1})}. \end{aligned}$$

Therefore, $$m - \gamma '_{SP}(0)$$ is:

D.4a $$\begin{aligned} m - \gamma _{SP}'(0)&= \frac{-(k_{-1}+k_2+k_1e_0)}{2k_{-1}} + \frac{k_2k_1e_0}{k_{-1}(k_1e_0+k_{-1})} \end{aligned}$$

D.4b $$\begin{aligned}&\quad + \frac{1}{2k_{-1}}\sqrt{(k_{-1} + k_2 + k_1e_0)^2 - 4k_1k_2e_0}, \end{aligned}$$

D.4c $$\begin{aligned}&= \frac{-(k_{-1}+k_1e_0)}{2k_{-1}} + \frac{k_2(k_1e_0 - k_{-1})}{2k_{-1}(k_1e_0+k_{-1})} \nonumber \\&\quad + \frac{1}{2k_{-1}}\sqrt{(k_{-1} + k_2 + k_1e_0)^2 - 4k_1k_2e_0}, \end{aligned}$$

D.4d $$\begin{aligned}&= \frac{-(k_{-1}+k_1e_0)}{2k_{-1}} + \frac{k_2(k_1e_0 - k_{-1})}{2k_{-1}(k_1e_0+k_{-1})} \end{aligned}$$

D.4e $$\begin{aligned}&\quad + \frac{1}{2k_{-1}}\sqrt{(k_{-1} + k_1e_0)^2 + k_2^2 + 2k_2k_{-1} - 2k_1k_2e_0}. \end{aligned}$$

Simplifying this expression, we want to show:

D.5 $$\begin{aligned} &  \frac{-(k_{-1}+k_1e_0)}{2k_{-1}} + \frac{k_2(k_1e_0 - k_{-1})}{2k_{-1}(k_1e_0+k_{-1})}\nonumber \\ &  \quad + \frac{1}{2k_{-1}}\sqrt{(k_{-1} + k_1e_0)^2 + k_2^2 - 2k_2(k_1e_0 - k_{-1})} > 0 \end{aligned}$$

or

D.6 $$\begin{aligned} \frac{1}{2k_{-1}}\sqrt{(k_{-1} + k_1e_0)^2 + k_2^2 - 2k_2(k_1e_0 - k_{-1})} > \frac{(k_{-1}+k_1e_0)}{2k_{-1}} - \frac{k_2(k_1e_0 - k_{-1})}{2k_{-1}(k_1e_0+k_{-1})}. \end{aligned}$$

The above expression is equivalent to:

D.7 $$\begin{aligned} &  (k_1e_0+k_{-1})\sqrt{(k_{-1} + k_1e_0)^2 + k_2^2 - 2k_2(k_1e_0 - k_{-1})} > \nonumber \\ &  \quad (k_1e_0+k_{-1})^2 - k_2(k_1e_0 - k_{-1}). \end{aligned}$$

When $k_2 < k_1e_0$, both sides are positive. Thus, we can prove that the above inequality is true by showing that the corresponding squared terms satisfy the same inequality. In simpler words, if $a, b > 0$ and $a^2 > b^2$, then $a > b$. Squaring both sides and simplifying, we can verify that:

D.8 $$\begin{aligned} &  (k_1e_0+k_{-1})^2((k_{-1} + k_1e_0)^2 + k_2^2 - 2k_2(k_1e_0 - k_{-1})) > \nonumber \\ &  \quad (k_1e_0+k_{-1})^4 + k_2^2(k_1e_0 - k_{-1})^2 - 2k_2(k_1e_0 - k_{-1})(k_1e_0+k_{-1})^2. \end{aligned}$$

Hence, the inequality $m - \gamma _{SP}'(0) > 0$ holds when $k_2 < k_1e_0$.

## Appendix E: Singular Perturbation Analysis of Large $k_{-1}$

In perturbation form, the Michaelis-Menten mass action equations with large $k_{-1}$ is

E.1a $$\begin{aligned} {\dot{s}}&= -k_1(e_0-c)s+\varepsilon ^{-1}\widehat{k}_{-1}c, \end{aligned}$$

E.1b $$\begin{aligned} {\dot{c}}&= \;\;\;k_1(e_0-c)s-k_2c-\varepsilon ^{-1}\widehat{k}_{-1}c, \end{aligned}$$

which admits the corresponding layer problem:

E.2a $$\begin{aligned} {\dot{s}}&= \varepsilon ^{-1}\widehat{k}_{-1}c, \end{aligned}$$

E.2b $$\begin{aligned} {\dot{c}}&= -\varepsilon ^{-1}\widehat{k}_{-1}c. \end{aligned}$$

The critical manifold is therefore *s*-axis and is normally hyperbolic and attracting. The projection matrix, $\pi ^s$, is given by

E.3 $$\begin{aligned} \pi ^s = \begin{pmatrix}1 & 1 \\ 0 & 0\end{pmatrix}. \end{aligned}$$

The Fenichel reduction is therefore

E.4 $$\begin{aligned} \begin{pmatrix}{\dot{s}}\\ {\dot{c}}\end{pmatrix} = \begin{pmatrix}1 & 1 \\ 0 & 0\end{pmatrix}\cdot \begin{pmatrix}-k_1(e_0-c)s+\varepsilon ^{-1}\widehat{k}_{-1}c\\ k_1(e_0-c)s-k_2c -\varepsilon ^{-1}\widehat{k}_{-1}c\end{pmatrix}\Bigg |_{c=0} = \begin{pmatrix}0\\ 0\end{pmatrix} \end{aligned}$$

which is trivial. Thus, one must appeal to higher-order terms in order to recover a non-trivial QSSA.

## Appendix F: QSS Approximations and Initial Conditions

This section briefly discusses how to determine appropriate initial conditions for quasi-steady-state approximations. For planar systems with a normally hyperbolic and attracting critical manifold, $S_0$, the two-dimensional stable manifold of $S_0$, denoted as $W^s(S_0)$, consists of one-dimensional manifolds called *fast* fibers. Each point $p_{{\mathcal {B}}}\in S_0$ is a base point, representing a steady-state (equilibrium) solution to the layer problem. Furthermore, for every base point in $S_0$, there exists a corresponding fiber ${\mathcal {F}}_0(p_{{\mathcal {B}}})$, which is the stable manifold of that base point:

$$\begin{aligned} \begin{aligned} {\mathcal {F}}_0(p_{{\mathcal {B}}}) =: W^s(p_{{\mathcal {B}}}), \quad W^s(S_0) = \bigcup _{p_{{\mathcal {B}}}\in S_0}{\mathcal {F}}_0(p_{{\mathcal {B}}}). \end{aligned} \end{aligned}$$

These base points serve as approximate initial conditions for quasi-steady-state reductions. Therefore, the task is to identify the unique base point that a given trajectory approaches during the transient (fast) phase of the reaction and then use this base point as the initial condition for the QSS reduction.

As an illustrative example, consider the Michaelis-Menten mass action system with small $k_2$. The perturbation form of the mass action system is:

F.1a $$\begin{aligned} \begin{aligned} {\dot{s}}&= -k_1(e_0-c)s+k_{-1}c,\quad {\dot{c}} = k_1(e_0-c)s-k_{-1}c-\varepsilon \widehat{k}_2c, \end{aligned} \end{aligned}$$

and the corresponding layer problem is

F.2a $$\begin{aligned} \begin{aligned} {\dot{s}}&= -k_1(e_0-c)s+k_{-1}c,\quad {\dot{c}} = k_1(e_0-c)s-k_{-1}c. \end{aligned} \end{aligned}$$

The critical manifold is:

F.3 $$\begin{aligned} S_0:= \left\{ (s,c)\in \mathbb {R}^2_{\ge 0}: c = \frac{e_0s}{K_S + s}\right\} \end{aligned}$$

and each base point, $p_{{\mathcal {B}}}$, can be represented as:

F.4 $$\begin{aligned} p_{{\mathcal {B}}} = (s,e_0s/(K_S+s)), \quad \forall s\in [0,s_0]. \end{aligned}$$

The typical initial condition for the Michaelis–Menten reaction mechanism is $(s,c)(0)=(s_0,0)$. As mentioned previously, the Fenichel reduction for small $k_2$ is:

F.5 $$\begin{aligned} \begin{pmatrix} {\dot{s}}\\ {\dot{c}} \end{pmatrix} = -\frac{k_2e_0 s(K_S+s)}{K_Se_0 + (K_S+s)^2}\begin{pmatrix} 1\\ \frac{K_Se_0 - K(K_S+s)}{(K_S+s)^2}\end{pmatrix}. \end{aligned}$$

A key question is whether we can equip (F.5) with the same initial condition, $(s,c)(0)=(s_0,0)$, and still expect the solution to the mass action system to converge to the solutions of (F.5) as $k_2\rightarrow 0$.

The answer is no. The reduction (F.5) approximates the dynamics on the slow manifold; it does not describe the dynamics of the approach to the slow manifold. While solutions to the mass action equations (F.1) capture both the fast and slow stages of the reaction, the QSS reduction (F.5) only describes the slow stage. Consequently, (F.5) requires a “modified” initial condition, $\tilde{x}_0=(\tilde{s},\tilde{c})$. This modified initial condition is the base point of the fast fiber containing $(s_0,0)$. In this example, the fiber containing $(s,c)(0)=(s_0,0)$ is:

F.6 $$\begin{aligned} {\mathcal {F}}_0(p_{{\mathcal {B}}}) =\{(s,c)\in \mathbb {R}^2_{\ge 0}: s+c=s_0\} \end{aligned}$$

and the modified initial condition is the intersection of ${\mathcal {F}}_0(p_{{\mathcal {B}}})$ with $S_0$:

F.7 $$\begin{aligned} \tilde{x}_0 = (s,e_0s/(K_S+s)), \quad \text {such that { s} satisfies:}\;\; s+ \frac{e_0s}{K_S+s}=s_0, \end{aligned}$$

which can be solved algebraically using the quadratic formula:

F.8 $$\begin{aligned} \tilde{s} = \frac{1}{2}\left( s_0-e_0-K_S+\sqrt{(s_0-e_0-K_S)^2+s_0K_S}\right) , \quad \tilde{c}= e_0\tilde{s}/(K_S+\tilde{s}). \end{aligned}$$

In summary, if we equip the reduction (F.5) with the modified initial condition $(s,c)(0)=(\tilde{s},\tilde{c})$, then the solution to this system will converge to the solution of the mass action system (F.1) (also equipped with the initial condition $(s,c)(0)=(\tilde{s},\tilde{c})$) as $k_2\rightarrow 0$.

Next, consider the case for small $e_0$, which is more complex than the case for small $k_2$. As previously mentioned, the critical manifold is the *s*-axis, but the QSS variety is actually the *c*-nullcline (a distinction not fully understood until recently; see Noethen and Walcher 2011 for details). The base points are $p_{{\mathcal {B}}}=(s,0)$, and the fibers are are more intricate (see Noethen and Walcher 2011, Sect. 3.1, for a discussion of the first integral of the layer problem associated with small $e_0$). However, since $(s_0,0)\in S_0$, we can equip the MM rate law,

F.9 $$\begin{aligned} {\dot{s}} = -\frac{k_2e_0s}{K_M+s} \end{aligned}$$

with the initial condition $s(0)=s_0$.

Initial conditions for the complex, *c*, can also be computed by analyzing the transient dynamics, but we will not delve into those details here. Interested readers can consult (Segel and Slemrod 1989) as well as Lax and Walcher (2020).

## Footnotes

1 Loosely speaking, solutions to the mass action system converge to solutions of the reduced system as $\varepsilon \rightarrow 0$ as long as initial conditions are sufficiently close to the critical manifold. Please visit “Appendix F” for details on the initial conditions of quasi-steady-state reductions.

2 In this context, *accuracy* refers to the ability of the reduction to approximate the flow on the slow manifold. We are choosing to ignore errors that arise in the approach to the slow manifold.

3 We assume $f(x,\varepsilon )$ is sufficiently smooth in both arguments.

4 Depending on $S_0$, $S_0^{\varepsilon }$ will either be attractive $W^u(S_0)=\emptyset \implies W^u(S_0^\varepsilon )=\emptyset$, repulsive $W^s(S_0)=\emptyset \implies W^s(S_0^\varepsilon )=\emptyset$, or will be of saddle type and come equipped with both stable and unstable manifolds.

5 Normal hyperbolicity of $S_0$ implies the algebraic and geometric multiplicity of $D_1f(x,0)$’s trivial eigenvalue is equal to *k*.

6 The operator $D_2$ denotes differentiation with respect to the second argument, *p*.

7 Here, $\widehat{e_0}$ is of unit magnitude and essentially denotes the unit of $e_0$. We adhere to a similar hat notation for the rest of the work.

8 In the (*x*, *y*) phase plane, if the upper fence lies above the lower fence, the set between the two fences is known as a *funnel*. Conversely, if the lower fence lies above the upper fence, the set between the two fences is called an *antifunnel* (Hubbard and West 1995). Refer to “Appendix B” for a brief synopsis on the theory of fences and anti-funnels and how it applies to the MM mechanism. See Hubbard and West (1995) for the detailed theory.

9 The graph of the *s*-nullcline is the set $\{(s,c) \in \mathbb {R}^2: c = e_0s/(K_S+s)\},$

10 Calder and Siegel (2008) define *I* as $[a, \infty )$ where $a > 0$ is arbitrary.

11 The equation is obtained via direct substitution of $c=\alpha _{cs}(s)$ into (2a).

12 The constants $\zeta _1,\zeta _2$ are introduced to enforce the compactness of $S_0^{(1)}$ and $S_0^{(2)}$.
